# Self-management practice and associated factors among asthmatic patients on follow-up care at public tertiary hospitals, south west, Ethiopia, 2022

**DOI:** 10.1371/journal.pone.0300589

**Published:** 2024-08-15

**Authors:** Girma Yadesa, Abebe Abera, Shamsedin Amme, Getahun Fetensa, Diriba Mulisa, Getachew Alemu

**Affiliations:** 1 Institute of Health, School of Nursing, Jimma University, Jimma, Ethiopia; 2 Department of Public Health, Institute of Health Sciences, Wollega University, Nekemte, Ethiopia; 3 Institute of Health Sciences, School of Nursing, Wollega University, Nekemte, Ethiopia; 4 School of Medical Laboratory, Institute of Health, Jimma University, Jimma, Ethiopia; Menzies School of Health Research, AUSTRALIA

## Abstract

**Introduction:**

Bronchial asthma is a major public health issue that affects patients, families, and communities worldwide. Despite the growing importance of self-management and its clear link to better health outcomes, the uptake and use of self-management among asthmatic patients is not well understood. Thus, the study aimed to determine the level of self-management practice and associated factors among asthmatic patients on follow-up care at public tertiary hospitals found in south-west Ethiopia.

**Method:**

An institutional-based cross-sectional study design was employed from August 1 to September 30, 2022. The study included all asthmatic patients on follow-up care at public tertiary hospitals found in south west Ethiopia, among whom 274 were interviewed. All asthmatic patients who were registered at the chronic follow-up unit of each hospital and fulfilled inclusion criteria were included and interviewed consecutively. The data were collected using a structured interviewer-administered questionnaire adapted from previous studies, entered into EpiData version 4.6, and exported to SPSS version 25 for analysis. Descriptive statistics were used to summarize the participant’s characteristics. Linear regression was used to identify variables associated with asthma self-management practice, and variables with a p-value of 0.05 were considered statistically significant. Finally, tables, graphs, and text were used to present the data.

**Result:**

Out of 274 interviewed asthmatic patients, 45.26% 95% CI (39, 51) had good asthma self-management practices. Advancing in age (ß = -0.009, P = 0.043), being a smoker (ß = -0.346, 95%, P = 0.03, being alcohol drinker (ß = -0.217, P = 0.001), having depression (ß = -0.038, P = 0.005), having anxiety (ß = -0.029, P = 0.02) and having social support (ß = 0.022, P<0.001) were identified as factors affecting asthma self-management practice.

**Conclusion:**

The finding revealed that four of every nine asthmatic patients had good asthma self-management practices. Age, alcohol consumption, smoking, anxiety, depression, and social support were significantly associated with asthma self-management practice. Ongoing self-management support and collaborative target interventions aimed at improving asthma self-management practices and identified factors are very crucial.

## Introduction

Bronchial asthma is a chronic, reversible airway obstruction that causes difficulty of breathing due to the constriction of the bronchi and mucus secretions [1]. Nowadays, bronchial asthma is a serious public health issue that has an impact on individuals, families, and communities all over the world. It is a leading cause of avoidable suffering that can be avoided by providing appropriate self-management information to asthma patients [2]. Adhering to ordered treatments, avoiding triggers, consulting a health care provider, promoting healthy lifestyle, and identifying the worsening of symptoms as well as responding to them correctly are self-performed activities [3,4].

Asthma self-management aims to achieve asthma symptoms control and maintain good activity levels despite the fact that there is no known cure for the condition. It contributes in lowering the likelihood of adverse effects from having asthma, severe complications, exacerbations, and ongoing airflow obstruction [5,6]. Further, self-management aims to improve health-related quality of life, promote patient independence, and decrease hospitalization rates, which then reduces nurses’ workload [7]. Progress in the management of chronic diseases like asthma has been slow, despite the fact that international aid and development programs have set targets for creating a more strong and efficient primary care health infrastructure [8]. Asthma was the 28thand 27th leading cause of disease burden globally and in low and middle-income countries (LMICs) on each nation’s sociological, financial, and healthcare systems respectively. According to the global asthma report, asthma is the 23rd leading cause of premature death globally and the 31st in low and middle-income countries, with an estimated 100 million people affected by 2025 [9,10].

Although patient and family involvement in asthma management is recommended, over the past 40 years there has been a noticeably increased mortality, morbidity, and economic burden of asthma worldwide [11,12].The ultimate goal of both expert and self-management is to keep asthma under control. However, according to the Communicable Disease and Control (CDC) report, half of adults and over 40% of children have uncontrolled asthma, though a wide range of pharmaceutical and other therapies are available [13]. According to a World Health Organization (WHO) report, asthma affected 262 million people in 2019 and killed 45,500 people [14].

Regarding economic burden, uncontrolled asthma has an annual direct and indirect cost of approximately $80 billion in the United States, which includes medical costs, lost productivity at work and school, and fatalities [15]. In the United Kingdom, there were 6.3 million primary care visits, 100,000 hospital admissions, 1800 admissions to intensive care units, and 78.9 days per 1,000 adult employment absences per year [16]. In India, 51.5% of patients experienced two to four emergency visits, 25.5% experienced five to ten visits, and 10.5% experienced more than ten visits as a result of an asthma exacerbation in the course of a year [17].

In Africa, approximately 119.3 million people have asthma, which results in a significant number of early deaths and severe or uncontrolled cases [18,19]. In sub-Saharan Africa, the average annual direct cost of asthma care per patient was $368.4 (228), and the majority (87%) of this cost was attributed to medication costs [20].

In Ethiopia, a systematic review found that 71.67% of patients had uncontrolled asthma, which was primarily due to patient-related factors [21]. Another study conducted in the northern part of Ethiopia found that approximately 88.9% of asthma patients had a history of frequent asthma exacerbations, with an average of two exacerbations per year [22]. In Jimma, 64.5% of respondents have uncontrolled asthma, which is mainly associated with low monthly income [23].

In conclusion, the study is required for a number of reasons. First, poor asthma control is widespread worldwide, despite the fact that the ultimate goal of a global initiative for managing asthma is to reduce symptoms and improve quality of life. Second, the importance of asthma self-management is highlighted by the global asthma report, which forecasts a 100 million increase in asthma cases by 2025. A systematic review in Ethiopia found that uncontrolled asthma, which raises the risk of morbidity, accounts for a considerable proportion of the country’s asthma cases. Finally, the majority of the existing literature focuses primarily on asthma self-management knowledge rather than practice. Hence, the study aimed to assess self-management practice and associated factors among asthmatic patients on follow up care at public referral hospitals in south west Ethiopia.

## Material and methods

### Study design, setting and period

A facility based cross-sectional study was conducted from August 1 to September 30 among asthmatic patients on follow-up care. The study was conducted in three public tertiary hospitals found in the southwest part of Ethiopia, namely: Jimma Medical Center, Mizan Tepi University Teaching Hospital, and Mattu Karl Referral Hospital. Jimma Medical Center (JMC) is found in Oromia Region, Jimma zone, Jimma town. It is the largest tertiary hospital found in southwest, Ethiopia. In this hospital, about 129 asthmatic patients have received follow-up care. Mizan-Tepi University Teaching Hospital (MTUTH) is located in the Bench Sheko Zone, Southern Nations Nationalities and Peoples Region (SNNPR), southwest, Ethiopia. The hospital is located 585 kilometers from Addis Ababa. In this hospital, about 84 asthmatic patients have received follow up care. Mettu Karl Referral Hospital (MKRH) is another tertiary public hospital found in southwest, Ethiopia, Oromia region, Ilu-Ababor zone, Mattu town. It is located about 600 kilometers from Addis Ababa, and 68 asthmatic patients were registered for follow-up care.

### Population

All asthmatic patients who were on follow-up care at public tertiary hospitals in south-west, Ethiopia were the source population, while those whose age was greater than 18 years and who were available during data collection were the study population. All asthmatic patients whose age was ≥18 years and who had at least one follow-up or medication refill were included in the study. Those study participants with communication problems and severe mental illnesses were excluded from the study.

### Sample size determination and sampling procedure

The number of asthmatic patients who were registered and have been on follow-up care at Jimma Medical Center, Mizan Tepi University Teaching Hospital, and Mattu Karl Referral Hospital was 129, 84, and 68, respectively. Since the total number of patients on follow-up care was 274, all asthmatic patients who were registered at each hospital’s chronic follow-up clinic were included in the study. Then, all patients visiting the chronic follow-up clinics at each hospital were consecutively interviewed.

### Data collection tool and procedures

Data were collected using questionnaire adapted from previous studies done in Bangladesh, Ethiopia, and the American Lung Association (ALA) guideline [22,24,25] and finally it was modified into the context of the study. It consists socio-demographic characteristics, clinical and behavioral characteristics, triggering and relieving factors, asthma self-management practice. the hospital anxiety and depression scale [26], the multi-dimensional perceived social support scale [27] and knowledge of Asthma Self-Management Questionnaire[28].

The outcome variable is asthma self-management practice. It was measured using 13 items, and for each item, participants rated their self-management practice on a four-point scale ranging from one (never perform = 1) to four (always perform = 4). The total scores range from 13 to 52 and the higher the score, the better the asthma self-management practices. The mean value of the self-management practice questions was used to classify the participant’s self-management practices as either good or poor. Hence, participants who scored above or equal to the mean were considered as good asthma self-management practice whereas those who scored below the mean were considered as poor asthma self-management practice.

Psychological factors included anxiety and depression-related questions. It has fourteen items (anxiety = 7 and depression = 7), on which the participants rated anxiety and depression on a three-point scale (definitely = 3 to not at all = 0). The total score ranges from 0 to 21 and was categorized as normal, borderline, and high level based on the HADS cut-off point. Social support was measured using multi-dimensional perceived social support questions. It has 12 items on which the patients rate social support on a seven-point scale from "strongly disagree" to "strongly agree. The mean value of the social support questions was used to classify the participant’s level of social support as either good or poor. Hence, those who scored equal to or above the mean were considered as good social support, whereas those who scored below the mean were considered as poor social support. Knowledge about asthma self-management was assessed using asthma self-management knowledge questionnaires (KASQ). The questionnaire consisted of nine items, which participants answered as true or false. The mean value of the asthma self-management knowledge questions was used to classify the participant’s knowledge as either good or poor. Hence, participants who scored above or equal to the mean were considered as good asthma self-management knowledge, whereas those who scored below the mean were considered as poor asthma self-management knowledge. Smokers refers to those participants who are currently smoking any tobacco products, such as cigarettes, cigars, or rolled tobacco [29]. Alcohol drinkers are those who have consumed a drink containing alcohol in the past 12 months [30]. Chat chewers are those who chewed Khat within the past 30 days prior to the study [31].

Initially, the questionnaire was prepared in an English version and then translated into the local languages (Afaan Oromo and Amharic versions) by language experts. Then, depending on the official (working) language of the regions, Amharic and Afan Oromo versions of the questionnaire were used for data collection. Two BSc nurses for data collection and one MSc nurse for supervision were recruited from each hospital. In hospitals where there were no MSc nurses, one BSc nurse with extensive experience in chronic follow-up care was hired for supervision. The data was collected from the patient himself or herself during his or her follow-up care (appointment).A face-to-face interview was conducted using a structured interviewer-administered questionnaire by trained data collectors.

### Data quality assurance and analysis

The English version of the questionnaire was translated into the Amharic and Afan Oromo versions and retranslated to English by a multilingual language expert. Before actual data collection, both data collectors and supervisors from each hospital were oriented regarding the purpose of data collection and the contents of the questionnaire. The questionnaire was tested on 5% (14 patients) before actual data collection at Shanan Gibe general hospital in Jimma town to assess the clarity of the questionnaire. The data collection process was supervised on a daily basis, and any ambiguity or other problems encountered by the data collectors were resolved before the following week with the assigned supervisors and principal investigators in person or using the phone.

Data was cleaned, coded, and entered into Epi-data version 4.6 and exported to SPSS version 25 for analysis. First, descriptive statistics was carried out. Frequency distribution, percentages, means, and standard deviations were used to summarize the participants’ characteristics. The mean was used to categorize asthma self-management practice and knowledge as poor or good. To check normality, a P-P plot, a Q- Q plot, and a histogram were used, and the plot showed that the points generally followed the normal line with no strong deviations. To check the linearity, a scatter plot was used, and the plot showed the linear relationship between dependent and independent variables. Hosmer and Lemeshow’s goodness of fit test was used to check the adequacy of the final model (p = 0.179). The assumption of multicollinearity was checked (VIF_max_ = 1.22). Before regression analysis, dummy variables were prepared for categorical variables such as marital status, educational level, occupation, and severity of asthma. Then simple linear regression or bivariate analysis was done to select candidate variables for multivariable regression at a p-value <0.25 after checking assumptions. During multivariable regression analysis, all candidate variables were entered once, and variables with a greater p-value were removed until only significant variables were left in the model by the backward selection method. Unstandardized coefficients, p-values, and 95% CI were used to declare the association between asthma self-management practice and predictor variables. In multivariable analysis, a p-value of 0.05 was considered statistically significant. Finally, the results were summarized and presented in the form of tables, charts, and graphs (supplementary material).

An ethical clearance and approval letter were obtained from Jimma University, Institute of health ethical review board before data collection. A support letter was written to all study areas, and agreement was made. Before data collection, the aim of the study was explained to the patients, and informed written consent was obtained. Each participant was notified about their right to refuse or participate in the study and the confidentiality of the information gathered. The study participants were assured that they would not be penalized for not participating, and that their response to the question would not affect their care. Respondents were also informed that their responses to the questions would be grouped with other participants’ answers and reported as part of a research study.

## Result

### Socio-demographic characteristics of the study participants

Out of 281 study participants who were on chronic follow-up care at southwest public tertiary hospitals, 274 asthmatic patients were interviewed, giving a response rate of 97.5%. Out of the study participants, 142 (51.8%) were female. The mean age of participants was 44 (SD = 15.8), with a minimum age of 18 and a maximum age of 68. Out of the 274 study participants who were interviewed, about 142 (51.8%) were female. Among the study participants, 106 (38.7%) were Muslim followers, followed by orthodox (38.3%). Among study participants, 165 (60.2%) were urban dwellers (**Table 1)**.

**Table 1:** Sociodemographic characteristics of asthmatic patients on follow-up care at public tertiary hospitals, southwest Ethiopia, 2022.

Characteristics	Category	Frequency	Percent
Sex	Male	132	48.2
	Female	142	51.8
Religion	Protestant	58	21.2
	Orthodox	105	38.3
	Muslims	106	38.7
	Others^a^	5	1.8
Residence	Urban	165	60.2
	Rural	109	39.8
Marital status	Married	194	70.8
	Single	80	29.2
Educational status	Illiterate	65	23.7
	Write and read	27	9.9
	Primary	47	17.2
	Secondary	49	17.9
	Preparatory	25	9.1
	College	32	11.7
	Degree &above	29	10.6
Occupation	Civil servant	75	27.4
	House wife	75	27.4
	Self-employee	34	12
	Retired	12	4.4
	Merchant	30	11.2
	Farmer	36	13.2
	Others	12	4.4
Insurance membership	Yes	167	60.9
	No	107	39.1
Mean ± SD insurance membership(minimum, maximum) 2.15±0.902(1,6)			
Mean ± SD age (minimum, maximum) 44.73±15.77(18,86)			

^*a*^*Catholic*, *Wakefata; SD- Standard deviation; ETB-Ethiopian birr*.

### Clinical and Behavioral characteristics of the study participants

The mean duration of asthma illness since diagnosis was 10.68 (SD = 8.63) years, with a minimum duration of 1 year and a maximum duration of 42 years. Of the study participants, 209 (74.8%) have no family history of asthma, and 143 (52.2%) have no comorbid conditions. Among participants, 56 (20.45%) were alcohol drinker whereas, 96 (35%) chewed a khat (**Table 2)**.

**Table 2:** Clinical and behavioral characteristics of asthmatic patients on follow-up care at the public tertiary hospital, southwest Ethiopia, 2022 (n = 274).

Characteristics	Category	Frequency (n = 274)	Percent
Family history of asthma	Yes	69	25.2
	No	205	74.8
Comorbidity	Yes	131	47.8
	No	143	52.2
Type of the comorbidities	Heart disease	23	8.4
	Diabetes mellitus	20	7.3
	Liver disease	11	4.0
	Renal disease	14	5.2
	Hypertension	45	16.4
	HIV/AIDS	5	1.8
	Others^b^	13	4.7
History of asthma exacerbation in last one year	Yes	225	82.1
	No	49	17.9
History of hospitalization in last one year	Yes	132	48.2
	No	93	33.9
Severity of asthma	Mild	117	42.7
	Moderate	128	46.7
	Severe	29	10.6
Asthma education	Yes	138	50.4
	No	136	49.6
Alcohol	Yes	56	20.4
	No	218	79.6
Cigarette	Yes	26	9.5
	No	248	90.5
Khat	Yes	96	35
	No	178	65
Illness duration(year)	M ± SD(maximu,minimum) 10.68 ±8.63(1,42)		
Exacerbation number	M± SD(maximum,minimum) 2.36 ±1.20(1,6)		
*Hospitalization number*	*M± SD(maximum*,*minimum) 1*.*30 ± 0*.*49(1*,*3)*		

^*a*^ *Human immune virus/Acquired immune deficiency syndrome*

^*b*^*Neurologic diseases*, *cancer*, *peptic ulcer disease*, *Thyrotoxicosis*, *Rheumatoid Arthritis*, *eye problem*, *other psychiatric disorders*, *SD- Standard Deviation*.

### Asthma aggravating and relieving factors

**Fig 1:**
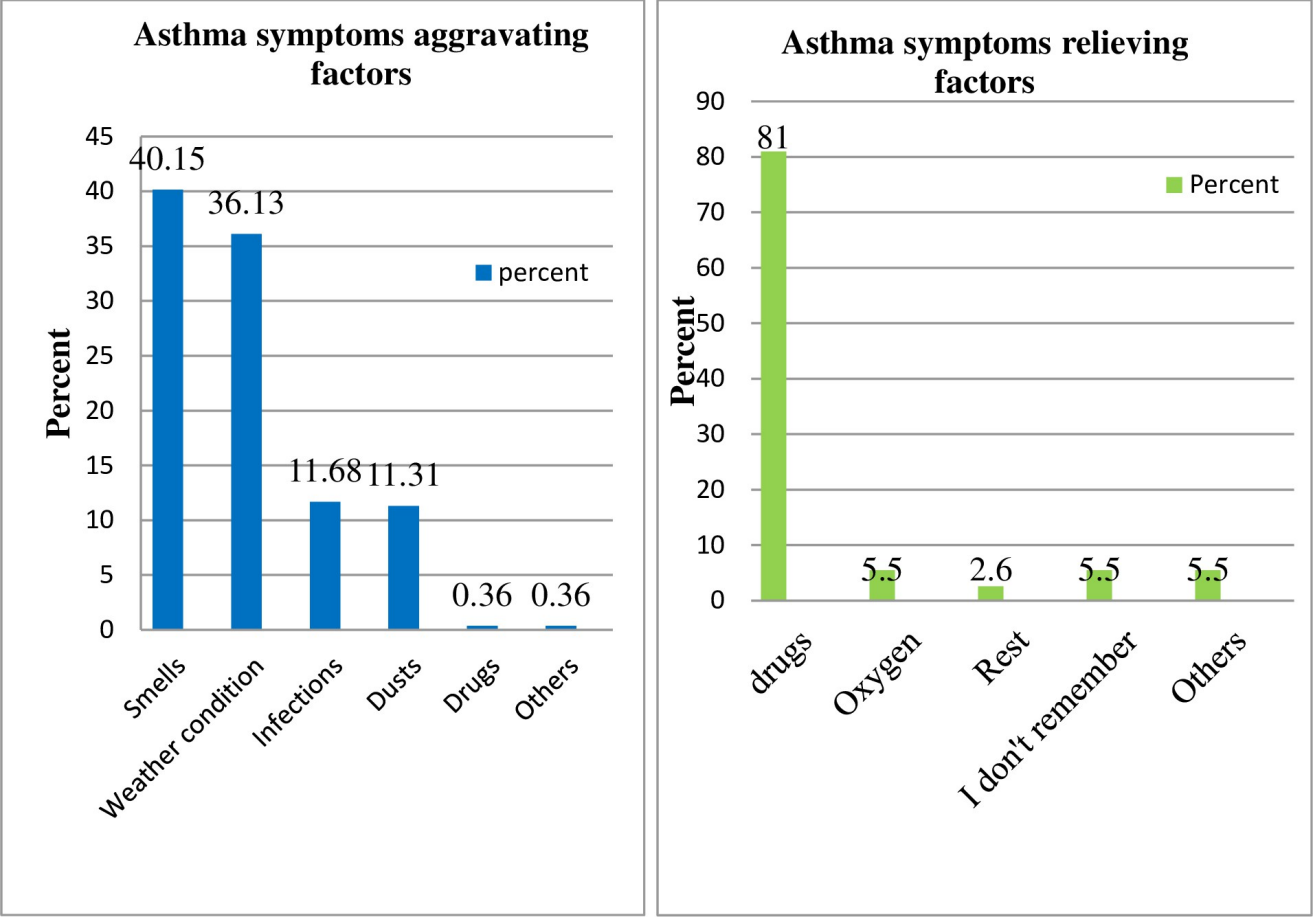
Aggravating and relieving factors among asthmatic patients on follow up care at public tertiary hospital.

As shown in Fig 1, the asthma symptoms of about 110 (40.15%) of the study participants were aggravated by smells from various sources, while weather conditions aggravated the asthma of 99 (36.13%). Regarding relieving factors, 81% of the study participants’ asthma symptoms were relieved by drugs.

### Descriptive statistics of some predictor variables (anxiety, depression and social support)

The mean of overall anxiety score in this study was 6.43 (SD = 3.081) with a minimum score of 0 and a maximum score of 18. Concerning the overall depression scores, the minimum and maximum score were 0 and 16, respectively, with a mean score of 4.93 (SD = 3.498). The mean score of asthma self-management knowledge was 4.98 (SD = 1.170) with a minimum and maximum score of 2 and 9, respectively. The mean score of overall social support of patients with asthma was 55.25 (SD = 10.107) whereas the minimum and maximum scores were 26 and 79 respectively (**Table 3).**

**Table 3:** Mean score of some predictor variables in the study of self-management practice and associated factors among asthmatic patients on follow up at public referral hospitals, south west Ethiopia,2022(n = 274).

Variables Mini		Max	Mean	SD
Over all anxiety score	0	18	6.43	3.081
Overall depression score	0	16	4.93	3.498
Total knowledge score	2	9	4.98	1.170
Overall social support	26	79	55.25	10.107

### Self-management practice of the study participants

Of the study participants, 109 (39.8%) frequently kept their medication schedules, while 25 (9.1%) always kept the schedule. Regarding controller drugs, about 147 (53.6%) of them were taking them frequently as prescribed by their physician, while 9 (3.3%) of them were never took as prescribed. Concerning keeping houses free of asthma triggers, about 119 (43.4%) and 24 (8.8%) kept houses free of triggers frequently and always, respectively. Concerning emotional control, about 107 (39.1%) of them sometimes tried to control their emotions, and 34 (12.4%) of them controlled them always. Of the study participants, 111 (40.5%) kept frequent track of how often they had asthma symptoms and how bad they were. Concerning seeing and asking health professionals, 128 (46.7%) of them sometimes saw health professionals even when they were well, while 124 (45.3%) of them were sometimes asked what they didn’t understand. Among respondents, about 109 (39.8%) of them frequently identified their symptoms as meaning they should get help right away **(Table 4).**

**Table 4:** Self-management practice among adult asthmatic patients on chronic follow-up at public tertiary hospitals, south west Ethiopia, 2022(n = 274).

Statements	Never	Sometimes	Frequent	Always
How often keep schedule of your medicine	37(13.5%)	103(37.6%)	109(39.8%)	25(9.1)
How often you take preventer drug as prescribed by health care provider	9(3.3%)	41(15%)	147(53.6%)	77(28.1%)
How often do you keep track of frequency of asthma symptoms	44(16%)	95(34.7%)	100(36.5%)	35(12.8%)
How often do you keep house free of asthma triggers	24(8.8%)	83(30.3%)	119(43.4)	48(17.5%)
How often do you take quick-relief medications before exercise/ activity	106(38.7%)	124(45.3%)	32(11.7%)	12(4.4%)
How often do you avoid activities because of asthma	38(13.9%)	109(39.8%)	93(33.9%)	34(12.4%)
How often do you try not to let emotions get out of control	35(12.8%)	107(39.1%)	98(35.8%)	34(12.4%)
How often do you keep track of how often you have asthma symptoms and how bad they are	42(15.3%)	74(27%)	111(40.5%)	47(17.2%)
How often do you avoid going places where you know people will be smoking	36(13.1%)	21(7.7%)	88(32.1%)	129(47.1%)
How often you see your healthcare provider regularly	15(5.5%)	109(39.8%)	108(39.4%)	42(15.3%)
How often do you see asthma health care provider even when you are well	98(35.8%)	128(46.7%)	41(15%)	7(2.6%)
How often do you ask your health care provider questions if you don’t understand something about your asthma	22(8%)	124(45.3%)	84(30.7%)	44(16.1%)
How often you identify your symptoms means you should get help right away	14(5.1%)	81(29.6%)	109(39.8%)	70(25.5%)

**Fig 2:**
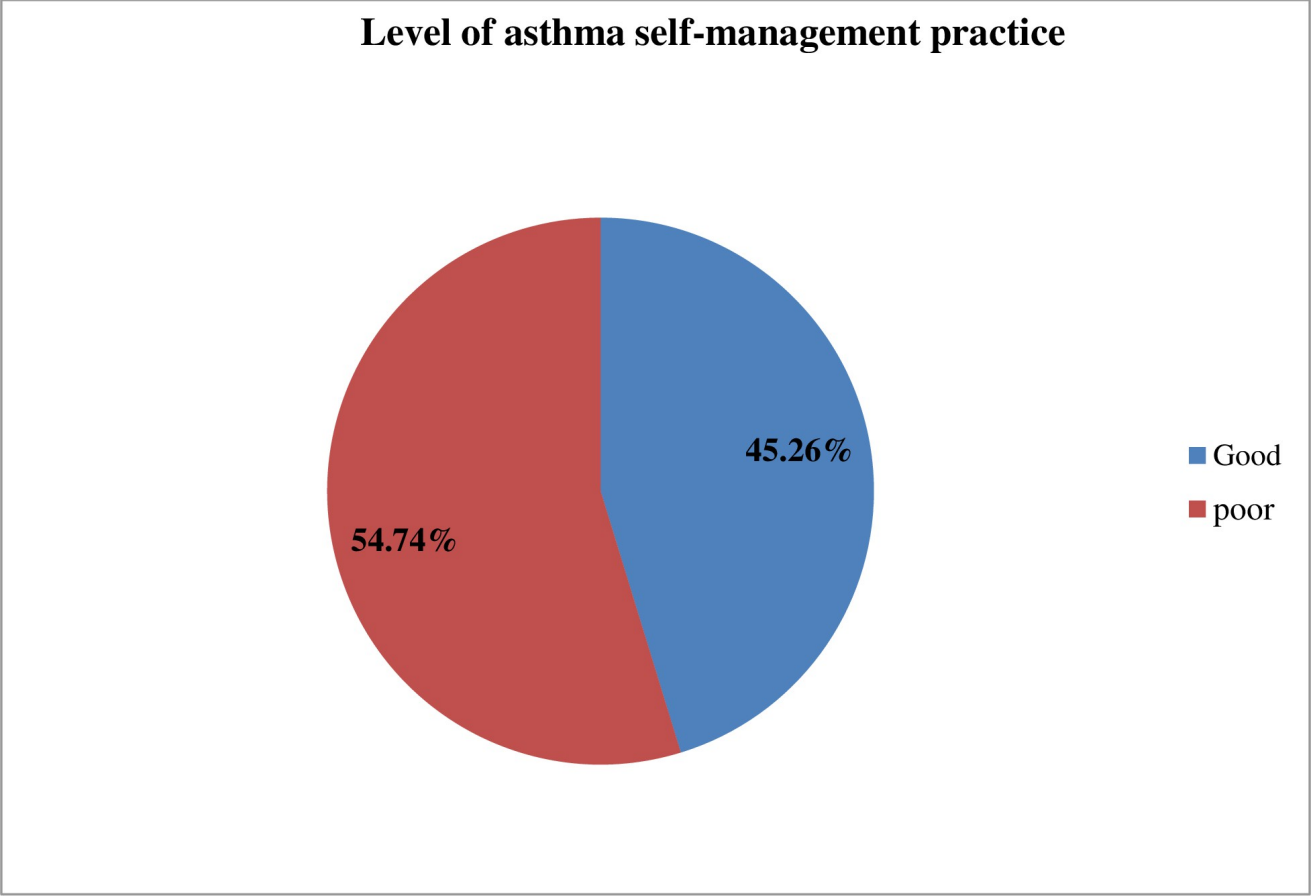
Level of asthma self-management practice among asthmatic patients on follow up care at public tertiary hospital.

As shown in Fig 2, the overall level of good self-management practice among asthmatic patients who were on follow-up at the southwest referral hospital was 45.26% 95%CI [39,51].

### Factors associated with asthma self-management practice

Out of eighteen possible significant variables in simple linear regression, six of them were identified as factors significantly associated with asthma self-management practice in multiple linear regression models. These were the respondent’s age, alcohol consumption, cigarette smoking, anxiety, depression, and social support.

Multivariable logistic regression analysis revealed that there is statistically significant negative association between the age of the respondent (ß = -0.009, 95% CI (-0.012, -0.005), P = 0.043) and self-management practice. This means that as age increased by one year, asthma self-management practice decreased by 0.009 while keeping other variables constant.

Additionally, patient behaviors like alcohol drinking (ß = -0.217, 95% CI (-0.338, -0.082), P = 0.001) and smoking cigarettes (ß = -0.346, 95% CI (-0.529, -0.180), P = 0.03) were negatively associated with asthma self-management practice. This implied that being an alcohol drinker or smoker decreased asthma self-management practice by 0.217 and 0.346, respectively, while controlling other variables.

Moreover, psychosocial factors such as anxiety (ß = -0.038, 95% CI (-0.06, -0.019), P = 0.02) and depression (ß = - 0.029, 95% CI (- 0.047, - 0.008), P = 0.005) were negatively associated with asthma self-management practice. This means, having anxiety and depression decrease asthma self-management practice by 0.038 and 0.029, respectively, while keeping other variables constant. On the other, hand the study identified the presence of a positive association between social support and asthma self-management practice (ß = 0.022, 95% CI (0.017, 0.029), P<0.001). This revealed that as social support increased by one unit, asthma self-management practice increased by 0.022 while keeping other variables constant. Generally, the model explains 63.3% of the variation in the dependent variable and the overall model is significantly useful in explaining responsiveness (adjusted R^2^ = 0.633, p<0.001 and F = 27.008) (**Table 5).**

**Table 5:** Multivariable linear regression analysis final model for the study of self-management practice and associated factors among asthmatic patients on follow up at public referral hospitals, south west Ethiopia, 2022(n = 274).

Predictors Variables	Unstandardized Coefficients		T	p-value	95.0% Confidence Interval for ß		Collinearity diagnostics	
	ß	Std. Error			Lower bound	Upper bound	Tolerance	VIF
age	-0.009	0.002	-3.647	0.043*	-0.013	-0.004	0.426	2.349
Illness duration	0.005	0.004	1.491	0.137	-0.002	0.013	0.626	1.597
Civil servant	0.062	0.072	0.865	0.388	-0.079	0.204	0.593	1.686
Others	-0.119	0.119	-1.001	0.318	-0.354	0.115	0.558	1.791
Smoking	-0.346	0.095	-3.654	0.03*	-0.533	-0.160	0.789	1.267
Chewing	-0.027	0.058	-0.461	0.645	-0.141	0.088	0.795	1.258
Alcohol	-0.217	0.067	-3.258	0.001*	-0.348	-0.086	0.857	1.168
Mild	0.083	0.053	1.559	0.120	-0.022	0.187	0.887	1.127
Insurance	0.014	0.057	0.251	0.802	-0.097	0.126	0.804	1.244
Anxiety	-0.038	0.012	-3.219	0.02*	-0.061	-0.015	0.471	2.123
depression	-0.029	0.010	-2.814	0.005*	-0.049	-0.009	0.484	2.067
Knowledge	0.013	0.023	-0.575	0.566	-0.058	0.032	0.864	1.157
Social support	0.022	0.003	6.530	<0.001**	0.016	0.029	0.519	1.928
Tertiary	0.125	0.093	1.345	0.180	-0.058	0.307	0.415	2.408
Secondery	0.089	0.068	1.314	0.190	-0.045	0.223	0.676	1.480
Comorbidity	-0.051	0.063	-0.806	0.421	-0.175	0.073	0.615	1.625

*Adjusted R*^*2*^ *= 0*.*633*, *Maximum VIF = 2*.*707 and p<0*.*001(F = 27*.*008)*

* = indicate significant value at (p<0.05) and

** = indicate highly significant value.

## Discussion

Self-management is an individual’s ability to manage the symptoms, therapy, physical and psychosocial implications, and lifestyle changes that come with having a chronic condition Self-management has been proposed to improve health-related quality of life, promote patient independence, and decrease hospitalization rate, hence reducing nurses’ workload, healthcare expense, and patient death rate [32]. This study’s findings revealed that 45.26% of the study participants had good asthma self-management practices. This is lower than the result of studies conducted in Bangladesh (62%), and Iran (57.7%) (45, 65). The difference might be due to sociodemographic variation, a measuring tool, health policy on chronic disease management, health care infrastructure, sample size, and probability sampling used by both Bangladesh and Iran studies. In contrast, the study finding is higher than the study conducted in Rwanda (33.8%) [33]. The possible explanation can be due to difference in measuring tool, sample size, and study settings included in the study. Additionally, the finding is higher than the study conducted in Gonder, Ethiopia (42.3%) [22]. The difference might be due to differences in study settings, sample size, and sampling method used.

This study again discovered that older patients have less self-management practice than younger patients. This is supported by several other studies in the USA, Taiwan, the United Arab Emirates, and Ethiopia [22,34–36]. This is due to the fact that aging results in molecular and cellular damage, which in turn results in reduced physical activity, mood disorders, social isolation, and an increased risk of chronic diseases. Additionally, the older age group can face challenges such as limited resources, increased cost of treatment, decreasing social support, and managing another chronic disease, which will result in poor asthma self-management practices during aging [37]. In contrast, studies in South Korea and Spain typically concluded that older age groups adhered to asthma self-management more consistently than younger age groups [38,39]. Possible explanations could be lower asthma severity perception, low benefit perception in the younger age group, and high health service utilization in the older age group.

A current study revealed that alcohol drinking was significantly associated with asthma self-management practices. This finding is supported by studies conducted in the USA, Rwanda and the northern part of Ethiopia [22,33,40]. Additionally, a study on chronic illnesses like diabetes mellitus supports the finding by concluding that current drinkers, starting with those who have one drink per day, were less likely to engage in good self-management practices than former drinkers [41]. This is due to the presence of sulfites in most consumable alcohols, which can trigger asthma attacks in some people. Furthermore, excessive and repeated alcohol consumption can harm mental activities such as acquiring, storing, retrieving, and using information given by educators [42,43].

The current study also found that smoking was significantly associated with self-management-practices. This finding is consistent with those of Tzu-Ting Huang and Claire Hayes-Watson [35,44]. This study concluded that smokers had lower asthma self-management. Smoking has been linked to changes in self-management behavior and perception that affect asthma control or adherence to self-management.

Moreover, in this study, anxiety and depression were identified as psychological factors that were significantly associated with asthma self-management practices. This finding was supported by several other studies conducted by Ilaria Baiardini, Sara l Leonard, Mangistu, and Abegaz [22,45–47]. These studies support the result by providing the conclusion that psychological problems primarily influence early recognition of their symptoms, how they manage their everyday lives, adherence to their medicine, and copies with the disease.

Lastly, social support was found to be a significant factor that affects asthma self-management practice. This is supported by studies conducted in Germany and the Nederland that concluded that social support affects asthma self-management activities such as trigger avoidance, acute symptom management, and communication with healthcare providers [48,49]. Other studies conducted in Taiwan, Malaysia, and Vietnam support this finding by concluding that self-management practice was affected by social stigma or embarrassment and fear of how other people perceive asthma and management [35,50,51]. Furthermore, the finding is also supported by a study conducted in the northern part of Ethiopia, which found that patients who have social support also have better asthma self-management practices [22]. This is because poor social support can result in a feeling of loneliness and isolation, which in turn lowers the mood and motivation of patients to participate in managing their chronic condition [42].

### Limitation of the study

The study has the following limitations: First, the study only showed self-reported self-management practice rather than the observation or actual practice. Second, there might be a recall bias and potential bias from socially acceptable responses, where people are reluctant to admit socially unacceptable behaviors such as smoking, drinking, or khat chewing in order to make a better impression.

## Conclusion and recommendation

In this study, four of every nine asthmatic patients had good asthma self-management practices. Furthermore, the respondent’s age, drinking alcohol, smoking, depression, anxiety, and social support were identified as factors significantly associated with asthma self-management practice. Hence, due attention should be given to identified factors while caring for patients with asthma. The inclusion of health care providers and rigorous study designs is recommended to evaluate the correlation between asthma self-management and associated factors.

## Supporting information

S1 File (DOCX)

## Abbreviations

BSc: Bachelor of Science
CDC: Communicable disease center
CI: Confidence Interval
LMIC: Lower Middle-Income Country
MSc: Master of Science
SD: Standard Deviation
SPSS: Statistical Package for Social Science
