# Supplementary material for: Self-management practice and associated factors among asthmatic patients on follow-up care at public tertiary hospitals, south west, Ethiopia, 2022
Source: PLoS One. 2024 Aug 15;19(8):e0300589. doi: 10.1371/journal.pone.0300589 (PMC11326582; doi:10.1371/journal.pone.0300589)
Supplement: S1 File — (DOCX) [file pone.0300589.s001.docx]

Table 10 Simple linear regression analysis showing lists of candidate variables for multivariable linear regression in the study of self-management practice among asthmatic patients on follow-up at public referral hospitals in south west Ethiopia,2022(n=274).

| Predictor variables | Unstandardized coefficient | | t | 95% CI for ß | | P-value |
| --- | --- | --- | --- | --- | --- | --- |
|  | **ß** | **Std. error** |  | **Lower** | **Upper** |  |
| Age | -0.021 | 0.002 | -9.149 | -0.025 | -0.016 | 0.001* |
| Sex(female) | -0.054 | 0.082 | -0.664 | -0.215 | 0.106 | 0.507 |
| Residence(rural) | -0.094 | 0.083 | -1.136 | -0.258 | 0.069 | 0.257 |
| Marital status(married) |  |  |  |  |  |  |
| Single | 0.091 | 0.103 | 0.879 | -0.112 | 0.293 | 0.380 |
| Education(illiterate) |  |  |  |  |  |  |
| Primary | 0.119 | 0.113 | 1.060 | - 0.102 | 0.341 | 0.290 |
| Secondary | 0.457 | 0.098 | 4.681 | 0.265 | 0.650 | 0.00* |
| Tertiary | 0.622 | 0.103 | 6.039 | 0.419 | 0.824 | 0.00* |
| Occupation(house wife) |  |  |  |  |  |  |
| Civil servant | 0.367 | 0.106 | 3.466 | 0.159 | 0.576 | 0.001* |
| Self-employee | 0.034 | 0.139 | 0.244 | -0.239 | 0.306 | 0.808 |
| Retired | -0.239 | 0.202 | -1.138 | -0.627 | 0.168 | 0.256 |
| Merchant | -0.071 | 0.150 | -0.472 | -0.366 | 0.224 | 0.637 |
| Farmers | 0.009 | 0.136 | 0.065 | -0.258 | 0.276 | 0.948 |
| Others | 0.471 | 0.155 | 3.045 | 0.166 | 0.775 | 0.003* |
| Insurance(has insurance) | 0.175 | 0.083 | 2.115 | 0.012 | 0.338 | 0.035* |
| Income(>1000 ETB) |  |  |  |  |  |  |
| >500ETB | -0.361 | 0.098 | -3.682 | -0.555 | -0.168 | 0.001* |
| 500-1000ETB | -0.483 | 0.093 | -5.179 | -0.667 | -0.300 | 0.001* |
| Family history (No) | -0.080 | 0.094 | -0.854 | -0.265 | 0.105 | 0.394 |
| illness duration | -0.018 | 0.005 | -3.942 | -0.027 | -0.009 | 0.001* |
| Comorbidity | -0.625 | 0.072 | -8.622 | -0.767 | -0.482 | 0.020* |
| Number of exacerbation | -0.028 | 0.037 | -0.744 | -0.101 | 0.046 | 0.458 |
| Number of hospital visit | -0.099 | 0.114 | -0.871 | -0.324 | 0.126 | 0.385 |
| Severity(moderate) |  |  |  |  |  |  |
| Mild | -0.339 | 0.137 | -2.469 | -0.610 | -0.069 | 0.014* |
| Severe | -0.002 | 0.085 | -0.023 | -0.170 | 0.166 | 0.982 |
| Alcohol drinking(No) | -0.528 | 0.097 | -5.467 | -0.718 | -0.338 | 0.000* |
| Smoking(No) | -0.751 | 0.131 | -5.715 | -1.010 | -0.492 | 0.000* |
| Khat user(No) | -0.368 | 0.083 | -4.445 | -0.530 | -0.205 | 0.001* |
| Asthma education(No) | 0.046 | 0.081 | 0.559 | -0.115 | 0.206 | 0.577 |
| Anxiety | -0.135 | 0.010 | -12.982 | -0.156 | -0.115 | 0.000* |
| Depression | -0.114 | 0.009 | -12.041 | -0.132 | -0.095 | 0.001* |
| Knowledge | 0.072 | 0.035 | 2.070 | 0.004 | 0.140 | 0.039* |
| Social support | 0.521 | 0.037 | 14.161 | 0.449 | 0.594 | 0.000* |

KEY: ***-** indicates p-value <0.25, ß-regression coefficient, CI-confidence interval, ETB-Ethiopian birr, VIF-variance inflation factor, ^a^wakefata, catholic; the categories in the brackets are reference categories.
